# Digenic inheritance of mutations in *SPG7* and *AFG3L2* causes motor neuron and cerebellar disorders

**DOI:** 10.1186/s12916-026-04805-z

**Published:** 2026-03-24

**Authors:** Mehrdad A. Estiar, Eric Yu, Parizad Varghaei, Jay P. Ross, Setareh Ashtiani, Andrew N. Bayne, Giulia Coarelli, Dagmar Timmann, Thomas Klockgether, Danique Beijer, David Mengel, Marie Coutelier, Project MinE ALS Sequencing Consortium, Patrick A. Dion, Oksana Suchowersky, Claire Ewenczyk, Cyril Goizet, Giovanni Stevanin, Philip Van Damme, Ammar Al-Chalabi, Stephan Zuchner, Matthis Synofzik, Jan H. Veldink, Jean-Francois Trempe, Alexandra Durr, Guy A. Rouleau, Ziv Gan-Or

**Affiliations:** 1 Department of Human Genetics, McGill University, Montréal, Québec Canada; 2 The Neuro (Montreal Neurological Institute-Hospital), McGill University, Montréal, Québec Canada; 3 Broad Institute of Harvard and MIT, Cambridge, MA USA; 4 Department of Psychiatry, University of Ottawa, Ottawa, ON Canada; 5 Alberta Children’s Hospital, Medical Genetics, Calgary, Canada; 6 Department of Pharmacology and Therapeutics, McGill University, Montréal, Québec Canada; 7 Centre de Recherche en Biologie Structurale (CRBS), McGill University, Montréal, Québec Canada; 8 Sorbonne Université, Paris Brain Institute - ICM, Inserm, CNRS, AP-HP, and Reference Center for Rare Diseases «Neurogenetics», Department of Medical Genetic, AP-HP, Hôpital Pitié-Salpêtrière, Paris, France; 9 Department of Neurology and Center for Translational Neuro- and Behavioral Sciences, University Hospital Essen, University of Duisburg-Essen, Duisburg, Germany; 10 German Center for Neurodegenerative Diseases (DZNE), Bonn, Germany; 11 Department of Neurology, University Hospital Bonn, Bonn, Germany; 12 Division of Translational Genomics of Neurodegenerative Diseases, Hertie-Institute for Clinical Brain Research and Center of Neurology, University of Tübingen, Tübingen, Germany; 13 German Center for Neurodegenerative Diseases (DZNE), Tübingen, Germany; 14 Department of Neurology and Neurosurgery, McGill University, Montréal, Québec Canada; 15 Departments of Medicine (Neurology) and Medical Genetics, University of Alberta, Edmonton, Canada; 16 Reference Center for Rare Diseases «Neurogenetics», Department of Medical Genetic, UMR 5287, Bordeaux University Hospital, and Univ. Bordeaux, CNRS, INCIA, NRGenTeam, Bordeaux, France; 17 Department of Neurology, Neuromuscular Reference Center, University Hospitals Leuven, Louvain, Belgium; 18 Department of Clinical Neuroscience, King’s College London, London, UK; 19 Dr. John T. Macdonald Foundation, Department of Human Genetics and John P, Hussman Institute for Human Genomics, University of Miami, Miller School of Medicine, Miami, FL USA; 20 Department of Neurology, University Medical Center Utrecht, Utrecht, The Netherlands

**Keywords:** Cerebellar disorder, Motor neuron disorder, Lateral sclerosis, Ataxia, SPG7, AFG3L2

## Abstract

**Background:**

Biallelic *SPG7* mutations cause one of the most common forms of hereditary spastic paraplegia (HSP). Several reports have suggested that heterozygous *SPG7* variants may also play a role in HSP, but also in amyotrophic lateral sclerosis (ALS). However, it remains controversial whether heterozygous *SPG7* mutations are pathogenic on their own, or if other mechanisms are at play. We recently provided evidence for non-Mendelian inheritance in spastic paraplegia 7 (SPG7), as heterozygous carriers of *SPG7* mutations often also carried mutations in other disease-related genes, including *AFG3L2*, more frequently than expected by chance. Given that *SPG7* and *AFG3L2* encode interacting subunits of the mitochondrial m-AAA protease complex, we hypothesized that combined heterozygous mutations in these genes may act synergistically to disrupt mitochondrial function and contribute to disease. In this study, we aimed to examine whether digenic heterozygous mutations in *SPG7* and *AFG3L2* can lead to a spectrum of neurodegenerative disorders.

**Methods:**

We first analyzed genome and exome sequencing data of 6644 unrelated individuals including 4817 motor neuron disorder (MND) and ataxia patients and 1827 controls. We next analyzed an additional 18,748 exome data from rare disease cohorts to further examine the occurrence of variants in *SPG7* and *AFG3L2*.

**Results:**

Among the first 4817 MND and ataxia patients, we identified a total of 6 patients, 4 of whom were unrelated, who carried potentially pathogenic variants in both *SPG7* and *AFG3L2*, in contrast to none in 1827 unrelated controls. Further analysis of the 18,748 additional patients with rare disease, as well as a comprehensive literature review, identified 6 more patients, 5 of whom were unrelated, who had digenic mutations in *SPG7* and *AFG3L2*. In the two families we identified, digenic mutations in *SPG7* and *AFG3L2* perfectly segregated with the disease. The 12 patients reported here exhibited predominant signs of motor neuron and cerebellar involvement.

**Conclusions:**

Our findings demonstrate that digenic inheritance of concurrent heterozygous mutations in *SPG7* and *AFG3L2* may cause motor neuron and cerebellar disorders. Screening of the entire *SPG7* and *AFG3L2* genes in genetically undiagnosed cases of MND and spastic ataxia may help to increase the diagnostic yield.

**Supplementary Information:**

The online version contains supplementary material available at 10.1186/s12916-026-04805-z.

## Background

*SPG7* is the first identified gene in autosomal recessive hereditary spastic paraplegia (HSP), with biallelic *SPG7* mutations found in 62% of autosomal recessive HSP patients [1]. *SPG7* encodes paraplegin, a mitochondrial matrix protease localized in the inner membrane of mitochondria [2], where it assembles with homologous AFG3L2 (ATPase family gene 3-like 2) subunits and forms a hetero-oligomeric proteolytic complex (SPG7-AFG3L2) [3, 4]. This complex is crucial for the normal function of mitochondria and its impairment results in mitochondrial dysfunction [3, 5]. SPG7 can only assemble with AFG3L2 to form this complex, whereas homo-oligomeric complexes of AFG3L2 (AFG3L2-AFG3L2) also exist.

Biallelic *SPG7* variants have been reported in multiple neurological disorders in addition to spastic paraplegia 7 (SPG7; OMIM 607259), including cerebellar and spastic ataxia [6–11], parkinsonism [12, 13], isolated optic atrophy [14], progressive external ophthalmoplegia [15], limb dystonia [16, 17], early-onset optic neuropathy [18], and primary progressive multiple sclerosis [19]. Several reports suggested that heterozygous *SPG7* variants may also be pathogenic in neurological disorders, including HSP [14, 20], primary lateral sclerosis (PLS) [21, 22], and amyotrophic lateral sclerosis (ALS) [23, 24].

While heterozygous *SPG7* mutations have also been suggested to cause disease, we showed in a recent study that many of those who carried a heterozygous *SPG7* variant also carried other variants in HSP-related genes or in genes that may interact with SPG7 [25]. This observation may suggest that SPG7 could be involved in digenic inherited neurological disorders rather than a monogenic effect of heterozygous *SPG7* variants. One of the HSP patients that we identified carried heterozygous variants in both *SPG7* and *AFG3L2* [25]. Interestingly, a previous case report described a patient with early-onset optic atrophy, spastic ataxia, and parkinsonism who also had digenic heterozygous mutations in *SPG7* and *AFG3L2*; yet, prior to this study, no additional patients had been reported. The case for digenic inheritance is further strengthened by various animal models with dysfunction of both proteins showing early-onset axonal degeneration, prominent cerebellar degeneration with loss of Purkinje cells and parallel fibers, reactive astrogliosis and defective mitochondria [26, 27].

In the current study, we aimed to examine whether digenic heterozygous mutations in *SPG7* and *AFG3L2* may indeed cause a spectrum of diseases in which the corticospinal and spinocerebellar tracts are affected, by analyzing genetic data from 4817 MND and ataxia patients and 1827 controls, followed by additional analysis of 18,748 individuals with rare diseases. We identified a total of 12 individuals with concurrent heterozygous mutations in *SPG7* and *AFG3L2* (9 of which were unrelated), and none in controls.

## Methods

### Participants

We used 6644 next-generation sequencing (NGS) datasets (Additional file 1: Table 1) including 6183 genome and 461 exome sequencing data. It included 4564 unrelated ALS patients and 1827 unrelated control individuals. The recruitment of 4356 ALS patients and 1827 age- and sex-matched controls was performed through the Project MinE cohort and the participants, including both cases and controls, were primarily of European ancestry [28]. An additional 208 ALS patients were recruited across Québec, Canada. We also analyzed exome sequencing data from 253 ataxia patients in France, recruited as part of the SPATAX cohort. In addition to these datasets, we analyzed 12,407 NGS datasets of rare diseases from the Genesis cohort [29] as well as data from 1341 patients with neurodegenerative disorders from Dr. Synofzik’s lab in Germany and 5000 patients with neurodegenerative disorders seen by various neurologists throughout Germany, to find potential concurrent heterozygous variants in *SPG7* and *AFG3L2*. We performed an extensive literature search via PubMed using the keywords “SPG7” and “AFG3L2.” We examined 31 studies for co-occurring *SPG7* and *AFG3L2* variants carriers.

**Table 1:** Genetic and clinical characteristics of patients with digenic mutations in *SPG7* and *AFG3L2*

Patient’s ID	1	2	3A	3B	3C	4
**Cohort**	ALS	ALS	Ataxia	Ataxia	Ataxia	Ataxia
***SPG7* mutation** **(**NM_003119.4**)**	c.1529C > T: p.(Ala510Val)	Het CNV (chr16:89,593,197;Del)	c.1529C > T: p.(Ala510Val)	c.1529C > T: p.(Ala510Val)	c.1529C > T: p.(Ala510Val)	c.1529C > T: p.(Ala510Val)
***AFG3L2* mutation** (NM_006796.3)	c.2167G > A: p.(Val723Met)	c.1894C > T: p.(Arg632Ter)	c.2105G > A: p.(Arg702Gln)	c.2105G > A: p.(Arg702Gln)	c.2105G > A: p.(Arg702Gln)	c.1976C > T: p.(Ala659Val)
**Sex**	M	M	M	F	F	M
**Origin**	The Netherlands	UK	French	French	French	French
**Family history and consanguinity**	Sporadic, no consanguinity	Sporadic, no consanguinity	Affected mother and sister, no consanguinity	Affected son and daughter, no consanguinity	Affected mother and brother, no consanguinity	Daughter probably affected (gait difficulties), no consanguinity
**Age at onset range (years)**	65–70	50–55	45–50	45–50	30–35	10–15
**Age at exam range (years)**	65–70	50–55	45–50	80–85	50–55	65–70
**Pyramidal motor system**						
UL/LL spasticity	Yes/Yes	Yes/Yes	None/none	None/none	None/none	None/none
UL/LL weakness	Yes/Yes	Yes/Yes	None/none	None/none	None/none	None/none
Tendon reflexes (hyperreflexia)	Yes	Yes	Increased reflexes	Normal UL, decreased at ankles	Normal	Normal UL, brisk LL
Muscle atrophy	Yes	Yes	None	None	None	None
Plantar response	Flexor		Indifferent	Flexor	Flexor	Flexor
Muscle stiffness	Yes	Yes	No	No	No	No
Ankle clonus	No	NA	No	No	No	No
Gait and balance instability	No	NA	Yes	Yes	Yes	Yes
Cognitive deficits	No	No	No	No	No	Yes
Behavioral and psychiatric symptoms	No	No	No	No	No	No
Developmental delay	No	NA	No	No	No	No
Optic atrophy	No	NA			No	Yes
Decreased visual acuity	No	NA		Yes	No	No
Bladder dysfunction	No	NA	No	Yes	No	No
**Extrapyramidal motor system**						
Brady-/hypokinesia	No	NA	No	No	No	Yes
Dystonia	No	NA	No	Yes (ULL)	No	No
Tremor	No	NA	No	No	No	No
Dyskinesia	No	NA	No	No	No	No
Others	No	NA	No	No	No	Amimia, rigidity
**Spinocerebellar system**						
Oculomotor	No	No		Slow saccades, vertical and horizontal ophthalmoplegia	Saccadic pursuit, slow saccades	Horizontal gaze evoked-nystagmus, vertical and horizontal ophthalmoplegia
Dysarthria/dysphagia	Yes (pseudobulbar)	Yes	No/No	Yes/yes	No/No	Yes/no
Ataxia	No	No	Yes SARA 3/40	Yes	Yes SARA 8.5/40	Yes SARA15/40
Slurred speech	No	Yes	No	Yes	Yes	Yes
Saccadic pursuit	No	NA	NA	Yes	Yes	Yes
**Sensory system**						
Vibration sense	Normal	NA	Normal	Abolished	Normal	Mild decreased
Joint position sense	Normal	NA	Normal		Normal	Normal
Surface sensation	Normal	NA	Normal	Normal	Normal	Normal
Temperature discrimination	Normal	NA	Normal	Normal	Normal	Normal
**EMG/ENG**	LMN involvement in lumbosacral and cervical region according to revised El Escorial, in lumbosacral and cervical and thoracic regions according to Awaji	Consistent with ALS				Sensory axonal neuropathy
**Imaging**	Normal	NA	NA	NA	Cerebellar atrophy	Cortical, corpus callosum, and cerebellar atrophy
**Other symptoms**	No	NA	NA	Ptosis, unable to walk requiring wheelchair	ptosis	ptosis

Patient’s ID	5A	5B	6	7	8	9 [published study]
**Cohort**	NDD (ataxia)	NDD (ataxia)	NDD (ataxia)	NDD (ataxia)	Genesis (ataxia)	Spastic ataxia with optic atrophy and parkinsonism
***SPG7* mutation** **(**NM_003119)	c.1063A > T: p.(Lys355Ter)	c.1063A > T: p.(Lys355Ter)	c.1529C > T: p.(Ala510Val)	c.1045G > A: p.(Gly349Ser)	c.1529C > T: p.(Ala510Val)	c.(376 + 1_377-1)_(861 + 1_862-1)del: p.Glu127SerfsTer2
***AFG3L2* mutation** (NM_006796)	c.2093A > G: p.(Asp698Gly)	c.2093A > G: p.(Asp698Gly)	c.2101G > T: p.(Val701Leu)	c.2065 T > C: p.(Tyr689His)	c.1678del: p.(Ser560AlafsTer32)	c.1402C > T: p.(Arg468Cys)
**Sex**	M	M	F	F	M	F
**Origin**	Germany	Germany	Germany	Germany	French	Italian
**Family history and consanguinity**	Affected son	Affected father	Sporadic, parents second degree cousins	Affected grandmother, father (died at age 46 years) and brother	Sporadic, no consanguinity	Sporadic, no consanguinity
**Age at onset range (years)**	35–40	40–45	35–40	15–20	30–35	5–10
**Age at exam range (years)**	45–50	50–55	55–60	70–75	40–45	25–30
**Pyramidal motor system**						
UL/LL spasticity	None/none	None/none	Very mild/none	None/none	None/none	None/yes
UL/LL weakness	None/none	None/none	None/none	None/none	None/none	
Tendon reflexes (hyperreflexia)	Normal	Normal	Patellar jerk hypereflexia	Increased lower extremities	Increased biceps and finger	
Muscle atrophy	None	None	None	None	None	
Plantar response	Flexor	Flexor	Flexor	Flexor	Flexor	
Muscle stiffness	No	No	No	No	No	
Ankle clonus	No	No	No	Yes	No	
Gait and balance instability	Yes	Yes	Yes	Yes	Yes	Yes
Cognitive deficits	No	No	Possible-mild (mild CCAS)	No	No	Mild
Behavioral and psychiatric symptoms	No	Np	No	No	No	
Developmental delay	No	No	No	No	No	
Optic atrophy	No	No	No	No		Yes
Decreased visual acuity	No	Np	No	No	No	Yes
Bladder dysfunction	No	No	Mild (pollakisuria)	No		
**Extrapyramidal motor system**						
Brady-/hypokinesia	No	No	No	No	No	Yes
Dystonia	Yes (facial)	No	No	No	No	Yes
Tremor	No	No	Yes, cerebellar tremor (intention tremor)		No	
Dyskinesia	No	No	No	No	No	Yes
Others		No		No	No	Motor fluctuations
**Spinocerebellar system**						
Oculomotor	Yes, external ophthalmoparesis, gaze-evoked nystagmus	Yes, horizontal gaze paresis, ptosis	Yes, horizontal nystagmus, reduced fixation suppression of VOR	Yes, external ophthalmoparesis Oscillopia Bilateral ptosis	Yes, horizontal nystagmus Horizontal Ophthalmoplegia	
Dysarthria/dysphagia	Yes	Yes	Moderate/mild-moderate	No	Yes	Mild/none
Ataxia	Yes SARA (2016) 7.5/40 (2015) 8/40 (2014) 6.5/40 (2012) 4/40	Yes	Yes SARA (2017) 15/40 (2016) 14/40 (2014) 15/40 (2012) 13.5/40 (2011) 11/40 (2005) 13/40	Yes SARA (2006) 12/40 (2024) 12.5/40	Yes SARA (2015) 11.5/40 (2011) 20/40	Mild
Slurred speech	Yes	Yes	Yes	Yes	Yes	
Saccadic pursuit	Yes	Yes	Yes	No	Yes	
**Sensory system**						
Vibration sense	Normal	Normal	Normal	Decreased (4/8 ankle)	Decreased	
Joint position sense	Normal	Normal	Normal	Normal		
Surface sensation	Normal	Normal	Normal	Normal	Normal	
Temperature discrimination	Normal	Normal	Normal	Normal		
**EMG/ENG**	Not performed	Normal NCS sensory sural and motor peroneal nerve; EMG normal triceps, vastus, and tibial muscle	Normal	Slight reduced amplitudes motor tibial nerve; normal amplitude and velocity of sensory sural nerve		
**Imaging**	Isolated cerebellar atrophy	Not performed	Cerebellar atrophy	Isolated cerebellar atrophy	Cortical and vermian atrophy	Normal
**Other symptoms**		None reported	OCT thin retina		CCFS: 0.945 in 2011 Amimia	Marked bilateral thinning of retinal fiber layer and ganglion cell layer

*chr*, chromosome; *Het*, heterozygous; *CNV*, copy number variation; *M*, male; *F*, female; *UL*, upper limb; *LL*, lower limb; *EMG*, electromyography; *ENG*, electroneurography; *LMN*, lower motor neuron; *SARA*, scale for the assessment and rating of ataxia; *MOCA*, Montreal cognitive assessment; *CCAS*, cerebellar cognitive affective syndrome; *MRI*, magnetic resonance imaging; *OCT*, optical coherence tomography; *VOR*, vestibulo-ocular reflex; *NDD*, neurodegenerative disease; *NA*, not available

### Genetic analysis

DNA was purified from peripheral blood according to standard procedures. Details on genome and exome sequencing were previously described [25, 30]. For genome sequencing data, variant calls with < 15 × depth of coverage and a genotype quality < 97 were excluded. For exome sequencing data, variant calls with < 30 × depth of coverage, a genotype quality < 97 and genotyping frequency < 25% were excluded from the analysis. We did relatedness tests on MND patients and controls using KING [31] and GCTA [32], separately. GCTA detected more related samples (3rd degree or closer), along with all the detected individuals by KING and then we removed 15 genome sequencing datasets including 10 cases and 5 controls.

The initial selection of variants was based on exonic (except for synonymous) and splice-site variants in *SPG7* (OMIM 602783, NM_003119.4, GRCh37/hg19) and *AFG3L2* (OMIM 604581, NM_006796.3, GRCh37/hg19) with allele frequency < 0.01 in gnomAD v2.1.1. Then, the variants were interpreted using VarSome [33] and the Franklin clinical variant interpretation platform [34], in accordance with the guidelines of the American College of Medical Genetics and Genomics (ACMG) and the Association for Molecular Pathology [35]. The variants were classified as either benign (B), likely benign (LB), uncertain significance (VUS), likely pathogenic (LP), or pathogenic (P). We excluded variants that were classified as B and LB. Splice-site variants were defined as variants occurring within ± 3 nucleotides of exon–intron boundaries. Intronic variants located beyond this region (> 3 bp from the splice junction) were excluded from the analysis, as they are less likely to directly affect canonical splicing.

The Manta tool was applied to call structural variants (SVs) from genome sequencing data. Multiplex ligation-dependent probe amplification (MLPA) was performed using the MLPA SALSA kit (MRC-Holland; Probemix P213 HSP mix-2) on a subset of samples that went through exome sequencing to discover copy number variations (CNVs). In order to predict the presence of important domains and sites of the corresponding protein, we used InterPro [36]. A *P*-value less than 0.05 was considered statistically significant for all results using binomial test, Pearson chi-square, or Fisher’s exact test.

### Structural visualization and mutational analysis

AFG3L2-SPG7 heterohexamer models were generated using the AlphaFold3 server [37]. Briefly, full length AFG3L2-SPG7 and matrix domain AFG3L2 (a.a. 272–797)-SPG7 (a.a. 279–795) heterohexamers were generated by specifying a 3:3 stoichiometry with default AlphaFold3 parameters. The resulting complex was relaxed using AMBER with 2000 iterations, a tolerance of 2.39 kcal/mol and a stiffness of 10 kcal/mol Å [37]. To estimate the locations of nucleotide binding sites and metal binding, one chain of each AFG3L2 and SPG7 matrix domains was inputted to the AlphaFill database [38] and the highest confidence predictions containing Mg^2+^, ADP, and Zn^2+^ co-factors were downloaded as individual PDB files. The positioning of these co-factors was verified by comparison to the cryo-EM structure of the substrate-bound AFG3L2 homohexamer (PDB: 6NYY) [39]. These individual AFG3L2/SPG7 chains from AlphaFill were then re-aligned onto the AFG3L2-SPG7 hexamer model in PyMOL v2.5.4 to position the co-factors within the entire assembly. All mutations and residue interfaces were visualized using PyMOL and annotated in Adobe Photoshop and Illustrator. To assess clashes induced by patient mutations, mutagenesis was performed in PyMOL and all rotamers were manually inspected. To support this manual mutagenesis approach, the stabilizing or destabilizing effects of individual missense mutations were also probed using DynaMut2 [40] on the unrelaxed AFG3L2-SPG7 heterohexamer model. The predicted changes in Gibbs Free Energy (ΔΔG) from DynaMut2 were manually annotated onto the structural visualization.

## Results

### Concurrent *SPG7* and *AFG3L2* mutations

**Fig. 1:**
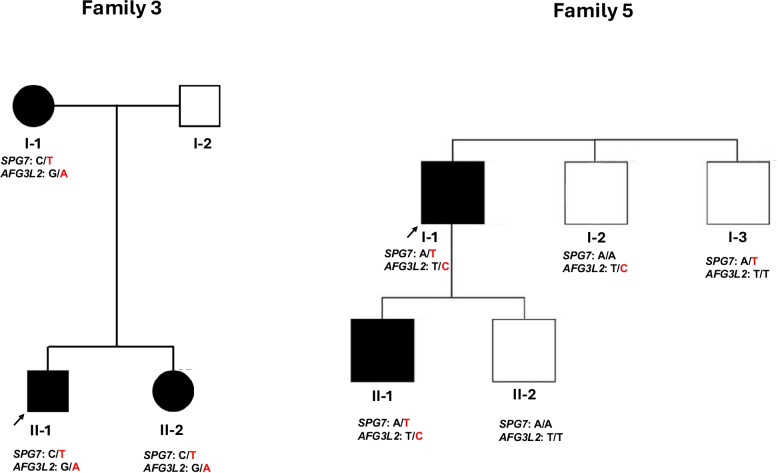
Pedigree of non-sporadic patients with available segregation data, demonstrating full segregation of the *SPG7* and *AFG3L2* variants

We analyzed 6644 exome and genome sequencing datasets from 4564 ALS patients, 253 ataxia patients, and 1827 control individuals to identify rare (< 1%) *SPG7* and *AFG3L2* variants. In the primary cohort, we found 4 index patients with P/LP co-occurring variants, whereas none were detected in controls (Table 1). One of these index cases was an affected mother with two affected children, all of whom carried both variants, demonstrating complete segregation within the family (Fig. 1). When rare VUS were included, we identified a total of six index patients carrying digenic *SPG7* and *AFG3L2* variants (Additional file 2: Table 2).

To further explore the spectrum of potentially pathogenic concurrent *SPG7* and *AFG3L2* variants, we expanded our analysis to exome sequencing data from 12,407 individuals with rare diseases in the Genesis cohort, as well as 6341 patients with neurodegenerative disorders (NDD). Additionally, a literature search identified previously reported cases with co-occurring *SPG7* and *AFG3L2* variants. Together, these efforts led to the identification of five additional unrelated patients with concurrent P/LP variants, bringing the total to 9 index patients, all presenting with symptoms of cerebellar disorders. Among these 9 patients (Table 1), two were ALS cases. One carried concurrent SNVs in both *SPG7* and *AFG3L2* and the other one carried a pathogenic nonsense *AFG3L2* SNV along with a heterozygous *SPG7* CNV. The remaining patients included four individuals from the ataxia cohort across France, four from NDD cohort across Germany, one from the Genesis rare disease cohort, and one previously reported case [27]*.* Including rare VUS, we identified a total of 15 patients carrying digenic *SPG7* and *AFG3L2* variants (Additional file 2: Table 2). Except for one of the *AFG3L2* SNVs, all other SNVs are either nonsense or occurred in the Peptidase M41 domain of AFG3L2 (IPR000642). The most common mutations in concurrent patients were *SPG7*:p.(Ala510Val) and *AFG3L2*:p.(Arg702Gln). In Families 3 and 5, comprising a total of 5 patients, digenic *SPG7* and *AFG3L2* variants co-segregated with disease (Fig. 1). In contrast, individuals carrying only one of the variants remained asymptomatic.

### Clinical characteristics of the digenic patients

Table 1 details the clinical characteristics of all patients, with gait difficulties being the main presenting symptom. Except for one patient, none of the concurrent patients came from consanguineous families, fewer than half were isolated cases (5/12), and the remainder presented with multigenerational disease. All cases were of European origin, and there were more men (7/12). The clinical presentation and progression were highly variable between patients. Cognition was not affected in most patients and mildly affected in some, and no developmental delay was reported in any of the patients. The sensory system was unaffected in most patients, but spinocerebellar signs were reported in most patients, especially oculomotor signs, and imaging demonstrated cerebellar atrophy in 5/8 patients in whom MRI was performed. Bladder function was normal in most patients.

### Frequency of non-digenic *SPG7* and *AFG3L2* variants in ALS patients

We did not find overrepresentation of P/LP *SPG7* or *AFG3L2* alleles separately in ALS patients vs. controls (*SPG7*: 103/9128 vs. 41/3654; *P* = 1; *AFG3L2*: 7/9128 vs. 2/3654; *P* = 1; Additional file 3: Table 3, Additional file 4: Table 4). Two ALS cases and one control individual carried biallelic P/LP *SPG7* variants. In both cases and controls, the most common P/LP *SPG7* allele was p.(Ala510Val), which was not significantly different between our ALS patients and controls (*SPG7*:p.Ala510Val: 54/9128, 0.59% vs. 22/3654, 0.60%,* P* = 1). After excluding the most frequent *SPG7* variant (despite all variants having MAF < 0.01), the number of P/LP alleles did not differ significantly between groups (*SPG7*: 49/9128 vs. 19/3654; *P* = 1; Additional file 3: Table 3). Similarly, the rare variant burden test in *SPG7* and *AFG3L2* using the Project MinE data browser (http://databrowser.projectmine.com/) also did not show a significant association (*SPG7 P* = 0.59; *AFG3L2 P* = 0.97; Additional file 1: Fig. S1). To assess the co-occurrence of rare variants in *SPG7* and *AFG3L2*, a binomial test was performed [41]. The analysis compared the occurrence of variants in 4564 ALS patients and 1827 controls. In the ALS group, 120 individuals carried rare *SPG7* variants and 14 carried rare *AFG3L2* variants, while in the control group, 43 individuals carried *SPG7* variants and 3 carried *AFG3L2* variants. The co-occurrence of these variants was observed in 3 ALS patients. This was significantly higher than expected under the null hypothesis of independent occurrence, with a *p*-value of 0.004, suggesting a significant association between the variants in ALS patients compared to controls.

### Structural analysis of AFG3L2/SPG7 hexamers and digenic mutations.

**Fig. 2:**
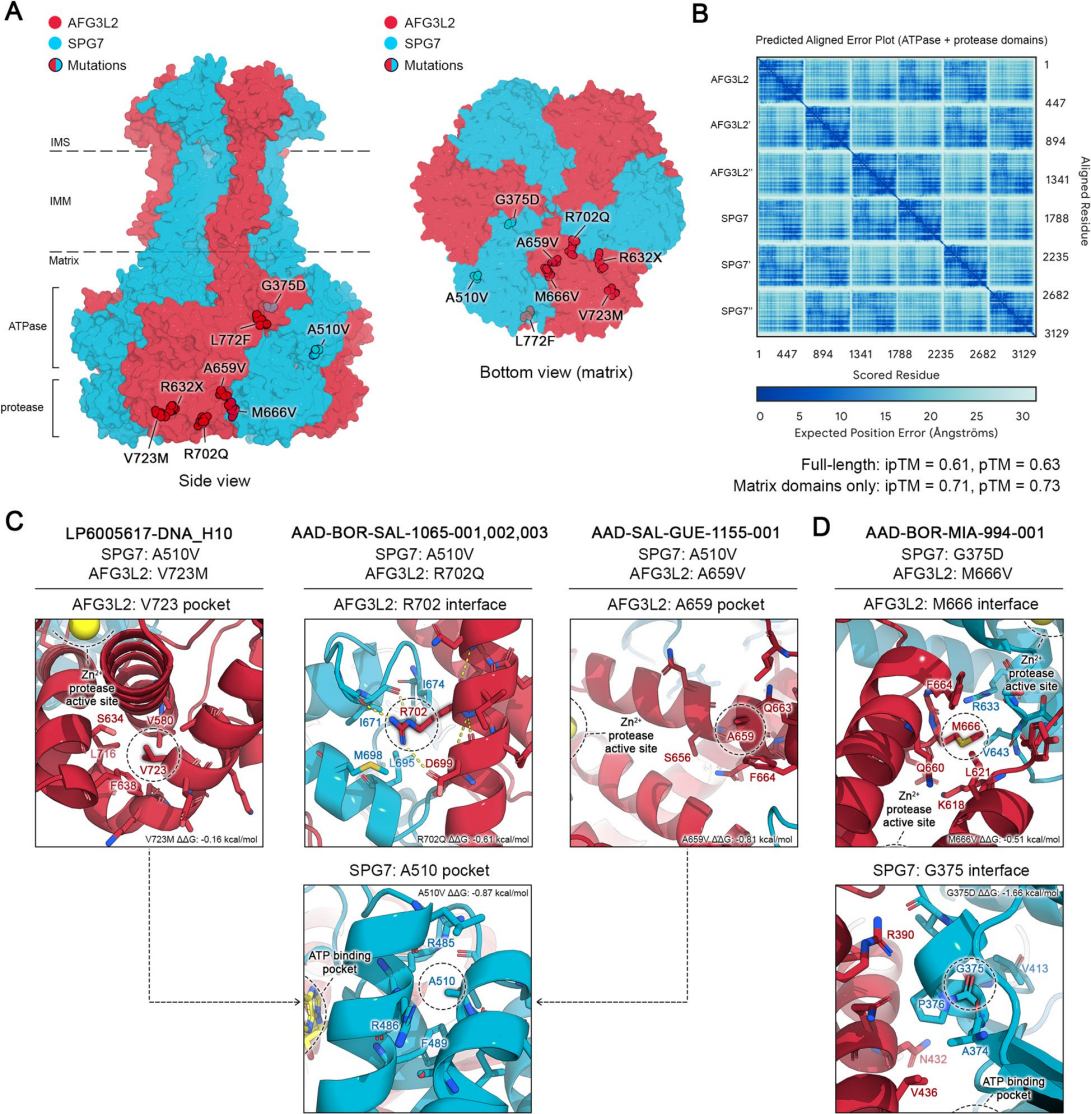
Structural analysis of *AFG3L2* and *SPG7* mutations. **A** Surface representation of full length AFG3L2-SPG7 heterohexamers as generated by AlphaFold3. Patient mutations were overlaid onto the surface structure and highlighted within the topology of the AFG3L2/SPG7 assembly. Unstructured N-terminal residues with low AlphaFold confidence scores (AFG3L2 a.a. 1–125 and SPG7 a.a. 1–122) were omitted from the surface representation for clarity. All patient mutations were visualized except for insertion-deletion variants and copy number variations. **B** Predicted aligned error (PAE) plot of the matrix domain AFG3L2/SPG7 assembly from AlphaFold3. Predicted template modeling (pTM) and interface predicted template modeling (ipTM) scores for both full length and matrix complexes were manually annotated. **C** Structural visualization of selected digenic missense mutations, visualized as atomic interaction sites of the wild-type residue. Residues within 4–5 Å of the target residue were visualized in PyMOL as sticks. Polar contacts formed by Arg702 are depicted as dashed yellow lines. Residues were annotated in Adobe Illustrator. **D** Structural visualization of SPG7 Gly375 and Met666 interaction sites, as in C

To probe the structural consequences of these digenic mutations, we utilized AlphaFold3 to generate a high-confidence model of an AFG3L2-SPG7 heterohexamer (Fig. 2A, B) and inspected the interactions formed by each wild-type residue of digenic missense mutation pairs. Most of these variants were localized to the matrix-exposed portions of AFG3L2 and SPG7, which contain their ATPase and metalloprotease domains. First, we analyzed the most common digenic pair: *SPG7*:p.(Ala510Val) and *AFG3L2*:p.(Arg702Gln) (Fig. 2C). SPG7 A510 is located on a short α-helix (α7) which faces the backside of another α-helix (α6) that forms the SPG7 nucleotide binding site. A510V is predicted to sterically clash with R485 of α6, which could destabilize nucleotide binding. A510 is not located near any inter-subunit contacts, so it is unlikely to have direct effects on oligomerization or assembly. Conversely, AFG3L2 R702 is predicted to be located at an inter-subunit interface between AFG3L2 and SPG7, where it forms polar contacts with SPG7 I671 and AFG3L2 D699. As such, while R702Q is not predicted to sterically clash with its neighboring SPG7, it could alter these polar interactions to reduce SPG7 binding. The cryo-EM structure of homohexameric AFG3L2 [39] also displays an equivalent R704 in AFG3L2 which mediates similar inter-subunit contacts. As such, in vitro work will be useful to address how *AFG3L2* R702Q and *SPG7* A510V might differentially affect AFG3L2-SPG7 assembly compared to AFG3L2-AFG3L2. Two other patients with SPG7 A510V mutations also harbored *AFG3L2* V723M and A659V mutations, respectively. Both residues are located close to the catalytic Zn^2+^ binding site and are not predicted to form inter-subunit contacts. Instead, their mutations may subtly alter AFG3L2 substrate binding and/or protease activity, though neither V723M nor A659V are predicted to introduce severe steric clashes with the AFG3L2 helices that bind Zn^2+^ directly.

Next, we analyzed another set of digenic missense mutations from one patient: *SPG7* G375D and AFG3L2 M666V, both of which are located at inter-subunit interfaces (Fig. 2D). Briefly, SPG7 G375 is located immediately after one β-strand (a.a. 369–374), which is part of the β-sheet that binds nucleotides close to the AFG3L2-SPG7 interface. In our model, SPG7 G375D is predicted to clash with the adjacent SPG7 loop containing V413, which could alter loop dynamics to disrupt inter-subunit AFG3L2-SPG7 contacts and/or nucleotide binding. AFG3L2 M666 is located within the AFG3L2 protease domain, in a hydrophobic pocket that mediates inter-subunit contacts via the Zn^2+^ binding helices of AFG3L2 and SPG7. While M666V is not predicted to directly clash with nearby residues, it could still disrupt the packing of Zn^2+^ binding helices in both inter- and intra-subunit contexts. In support of this model, recombinant mutant M666V AFG3L2 homohexamers displayed reduced proteolytic and ATPase activity compared to wild type AFG3L2 [39]. Moreover, recombinant AFG3L2 M666R, a more severely disruptive mutation at the same interface also did not assemble into homohexamers, though the effect of M666V on complex assembly remains unclear. As such, the distinct effects of AFG3L2 M666V on SPG7 binding and proteolysis within the context of the AFG3L2-SPG7 heterohexamer require further studies. Given that the structural mechanisms which govern upstream AFG3L2/SPG7 assembly and folding also remain unclear, in vitro studies will be essential to clarify how these digenic mutations interact together within AFG3L2/SPG7 assemblies to affect their assembly, oligomerization, and/or activity.

## Discussion

Our results show that digenic inheritance of potentially pathogenic variants in *SPG7* and *AFG3L2* may cause a spectrum of motor neuron and spinocerebellar neurodegenerative disorders. Notably, the majority of *AFG3L2* mutations occur in the peptidase domain, indicating that these mutations may abolish proteolytic activity and do not allow the interaction of AFG3L2 with SPG7 subunits into hetero-oligomeric proteolytic complex in the inner mitochondrial membrane. The specific type and position of mutations may explain some of the heterogeneity in the phenotypic spectrum. For instance, the majority of ataxic patients carried *AFG3L2*:p.(Arg702Gln) and *SPG7*:p.(Ala510Val) single nucleotide mutations.

SPG7 performs its function by assembling with its homologous and biological counterpart, AFG3L2, forming a heterohexamer. Both *SPG7* and *AFG3L2* have the highest expression levels in the cerebellum compared to other regions of the brain (GTEx). Furthermore, they encode highly similar proteins with identical conserved domains. These observations may explain why either homozygous mutations or digenic heterozygous mutations in *SPG7* and *AFG3L2* may lead to the same disorders. Insertions or deletions (indels) in *SPG7* or *AFG3L2* can disrupt the formation and function of the hetero-oligomeric m-AAA protease complex by altering protein structure, stability, or expression levels. Deletions may cause haploinsufficiency, leading to insufficient subunits for complex assembly, while insertions could introduce aberrant sequences, resulting in misfolded or nonfunctional proteins. Such disruptions impair mitochondrial proteostasis, exacerbating dysfunction in digenic inheritance scenarios where pathogenic variants in both genes coexist.

Previous studies have suggested that heterozygous *SPG7* mutations may cause ALS [42, 43]. In our analysis of a large ALS cohort, the results suggest that heterozygous *SPG7* variants alone are unlikely to be sufficient to cause ALS. Given our current results on the digenic inheritance of *SPG7* and *AFG3L2*, we suggest that a likely explanation for previous reports is that carriers of heterozygous *SPG7* variants may also carry variants in *AFG3L2* or in other, yet-to-be-discovered genes. An additional explanation is that undetected structural variants in *SPG7*, which are not often detectable by conventional methods, may have contributed to these cases. Additionally, in these previous reports, the heterozygous *SPG7* variants were inherited from asymptomatic parents aged over > 75 in two of the reported families. Taken together, our results challenge the notion of autosomal dominant inheritance of *SPG7* in ALS.

We previously reported a high number of heterozygous P/LP *SPG7* alleles in HSP patients vs. controls [25]. In this case too, the observed overrepresentation of heterozygous *SPG7* variants in patients could be explained by unknown digenic inheritance and other reasons. One additional explanation is that our previous study was based on exome sequencing analysis, which is not capable of detecting some classes of disease-causing variants (e.g., structural variants or variants in intronic regions). For example, CNV analysis of the same data revealed the presence of additional *SPG7* CNVs in two heterozygous carriers of *SPG7* SNVs, showing that they were in fact biallelic carriers of *SPG7* mutations and not heterozygous carriers as we initially reported [25]. Furthermore, a deep intronic *SPG7* variant inducing the inclusion of a pseudoexon and premature stop sites was identified in a patient with heterozygous missense *SPG7* variant [44]. This variant and other deep intronic variants that may lead to similar effects cannot be detected with exome sequencing. Hence, re-examination and full screening of the entire *SPG7* gene are required for the genetic diagnosis of cerebellopathy patients with at least one detected pathogenic *SPG7* allele. This analysis can then be supplemented by comprehensive analysis for digenic inheritance with *AFG3L2* and other related genes.

Our study has some limitations. We did not perform functional analysis to examine the cellular interaction of SPG7 and AFG3L2 in relevant models, which will be required to fully understand the underlying mechanism. However, previous studies in mice and yeast showed digenic interaction of *spg7-afg3l2* and additive effect of *spg7* and *afg3l2* variants [26, 27], supporting our genetic-clinical findings. Moreover, this study focused solely on *SPG7* and *AFG3L2*, without examining other interacting genes such as *PHB*, *PHB2*, or *STOML2*. Therefore, additional digenic or oligogenic combinations within this network may have been overlooked. Moreover, some observed variant combinations could represent random co-occurrences rather than true biological interactions. Another limitation is that some variants identified in our patients, though very rare, are classified as VUS according to ACMG and require pathogenic validation. The predominant SCA28 phenotype underscores the complexity of digenic inheritance involving *SPG7* and *AFG3L2* variants, highlighting the need to consider both in genetic counseling, despite the current focus on the SCA28 variant for presymptomatic testing.

## Conclusions

Our results support a potential role for digenic inheritance involving *SPG7* and *AFG3L2* in cerebellopathy and corticospinal tractopathy. These findings highlight the importance of comprehensive genetic evaluation in individuals with ataxia and motor neuron disorders who carry a single *SPG7* variant. Further studies will be required to clarify the role of this interaction and its relevance for genetic evaluation and therapeutic exploration in *SPG7*-associated neurodegenerative disorders.

## Supplementary Information

Additional file 1: Table 1 - Subjects included in the study.

Additional file 2: Table 2 - Genetic and clinical characteristics of patients carrying rare P/LP/VUS digenic SPG7 and AFG3L2 variants.

Additional file 3: Table 3 - List of potentially pathogenic SPG7 variants identified in ALS patients and controls.

Additional file 4: Table 4 - List of potentially pathogenic AFG3L2 variants identified in patients and controls.

Additional file 5: Fig. 1 - Burden testing results based on the ProjectMinE genome sequencing dataset. The figure derived from Project MinE data browser and shows the exons (orange blocks) in SPG7 and AFG3L2 with the variants (triangles) that were observed in the genome sequencing dataset.

## Data Availability

All data used in this study are described in the Methods section. Data generated as part of this study are available from the corresponding author upon reasonable request.

## Abbreviations

HSP: Hereditary spastic paraplegia
ALS: Amyotrophic lateral sclerosis
SPG7: Spastic paraplegia 7
MND: Motor neuron disorder
NGS: Next-generation sequencing
ACMG: American College of Medical Genetics and Genomics
B: Benign
LB: Likely benign
VUS: Uncertain significance
LP: Likely pathogenic
P: Pathogenic
SV: Structural variant
MLPA: Multiplex ligation-dependent probe amplification
CNV: Copy number variations
NDD: Neurodegenerative disorders
