# Supplementary material for: Digenic inheritance of mutations in SPG7 and AFG3L2 causes motor neuron and cerebellar disorders
Source: BMC Med. 2026 Mar 24;24:283. doi: 10.1186/s12916-026-04805-z (PMC13134353; doi:10.1186/s12916-026-04805-z)
Supplement: Supplementary file 1 — Additional file 1: Table 1 - Subjects included in the study. [file 12916_2026_4805_MOESM1_ESM.docx]

**Supplementary Table 1** Subjects included in the study

| **Status** | **Cohort** | **Genome Sequencing** | **Exome Sequencing** | **Total** |
| --- | --- | --- | --- | --- |
| Controls | Project MinE + Dr. Rouleau’s lab | 1827 | - | 1827 |
| ALS | Project MinE + Dr. Rouleau’s lab | 4356 | 208 | 4564 |
| Ataxia | SPATAX cohort, screened in Dr. Durr’s lab | - | 253 | 253 |
| NDD | Dr. Synofzik’s lab | - | 1341 | 1341 |
| NDD | A Cohort, screened in Dr. Synofzik’s lab | - | 5000 | 5000 |
| Rare diseases | GENESIS | - | 12407 | 12407 |

ALS = amyotrophic lateral sclerosis; HSP = hereditary spastic paraplegia; MND = motor neuron disease, NDD = Neurodegenerative disorders
