# Supplementary material for: Digenic inheritance of mutations in SPG7 and AFG3L2 causes motor neuron and cerebellar disorders
Source: BMC Med. 2026 Mar 24;24:283. doi: 10.1186/s12916-026-04805-z (PMC13134353; doi:10.1186/s12916-026-04805-z)
Supplement: Supplementary file 2 — Additional file 2: Table 2 - Genetic and clinical characteristics of patients carrying rare P/LP/VUS digenic SPG7 and AFG3L2 variants. [file 12916_2026_4805_MOESM2_ESM.docx]

**Supplementary Table 2.** Genetic and clinical characteristics of patients carrying rare P/LP/VUS digenic *SPG7* and *AFG3L2* variants.

| **Patient’s ID** | **1** | **2** | **3A** | **3B** | **3C** | **4** | **5A** | **5B** |
| --- | --- | --- | --- | --- | --- | --- | --- | --- |
| **Cohort** | ALS | ALS | Ataxia | Ataxia | Ataxia | Ataxia | NDD (Ataxia) | NDD (Ataxia) |
| ***SPG7* mutation**  **(**NM_003119.4**)** | c.1529C>T:  p.(Ala510Val) | Het CNV (chr16:89593197;Del) | c.1529C>T:  p.(Ala510Val) | c.1529C>T:  p.(Ala510Val) | c.1529C>T:  p.(Ala510Val) | c.1529C>T:  p.(Ala510Val) | c.1063A>T:  p.(Lys355Ter) | c.1063A>T:  p.(Lys355Ter) |
| ***AFG3L2* mutation**  (NM_006796.3) | c.2167G>A:  p.(Val723Met) | c.1894C>T:  p.(Arg632Ter) | c.2105G>A:  p.(Arg702Gln) | c.2105G>A: p.(Arg702Gln) | c.2105G>A: p.(Arg702Gln) | c.1976C>T: p.(Ala659Val) | c.2093A>G:  p.(Asp698Gly) | c.2093A>G:  p.(Asp698Gly) |
| **Sex** | M | M | M | F | F | M | M | M |
| **Origin** | The Netherlands | UK | French | French | French | French | Germany | Germany |
| **Family history and consanguinity** | Sporadic, no consanguinity | Sporadic, no consanguinity | Affected mother and sister, no consanguinity | Affected son and daughter, no consanguinity | Affected mother and brother, no consanguinity | Daughter probably affected (gait difficulties), no consanguinity | Affected son | Affected father |
| **Age at onset range (y)** | 65-70 | 50-55 | 45-50 | 45-50 | 30-35 | 10-15 | 35-40 | 40-45 |
| **Age at exam range (y)** | 65-70 | 50-55 | 45-50 | 80-85 | 50-55 | 65-70 | 45-50 | 50-55 |
| **Pyramidal motor system** |  |  |  |  |  |  |  |  |
| UL/LL spasticity | Yes/Yes | Yes/Yes | None/none | None/none | None/none | None/none | None/none | None/none |
| UL/LL weakness | Yes/Yes | Yes/Yes | None/none | None/none | None/none | None/none | None/none | None/none |
| Tendon reflexes (hyperreflexia) | Yes | Yes | Increased reflexes | Normal UL, decreased at ankles | Normal | Normal UL, brisk LL | Normal | Normal |
| Muscle atrophy | Yes | Yes | None | None | None | None | None | None |
| Plantar response | Flexor |  | Indifferent | Flexor | Flexor | Flexor | Flexor | Flexor |
| Muscle stiffness | Yes | Yes | No | No | No | No | No | No |
| Ankle clonus | No | NA | No | No | No | No | No | No |
| **Gait and balance instability** | No | NA | Yes | Yes | Yes | Yes | Yes | Yes |
| **Cognitive deficits** | No | No | No | No | No | Yes | No | No |
| **Behavioral and psychiatric symptoms** | No | No | No | No | No | No | No | Np |
| **Developmental delay** | No | NA | No | No | No | No | No | No |
| **Optic atrophy** | No | NA |  |  | No | Yes | No | No |
| **Decreased visual acuity** | No | NA |  | Yes | No | No | No | Np |
| **Bladder dysfunction** | No | NA | No | Yes | No | No | No | No |
| **Extrapyramidal motor system** |  |  |  |  |  |  |  |  |
| Brady-/hypokinesia | No | NA | No | No | No | Yes | No | No |
| Dystonia | No | NA | No | Yes (ULL) | No | No | Yes (facial) | No |
| Tremor | No | NA | No | No | No | No | No | No |
| Dyskinesia | No | NA | No | No | No | No | No | No |
| Others | No | NA | No | No | No | Amimia, rigidity |  | No |
| **Spinocerebellar system** |  |  |  |  |  |  |  |  |
| Oculomotor | No | No |  | Slow saccades, vertical and horizontal ophthalmoplegia | Saccadic pursuit, slow saccades | Horizontal gaze evoked-nystagmus, vertical and horizontal ophthalmoplegia | Yes, external ophthalmoparesis, gaze-evoked nystagmus | Yes, horizontal gaze paresis, ptosis |
| Dysarthria/dysphagia | Yes (pseudobulbar) | Yes | No/No | Yes/yes | No/No | Yes/no | Yes | Yes |
| Ataxia | No | No | Yes  SARA 3/40 | Yes | Yes  SARA 8.5/40 | Yes  SARA15/40 | Yes  SARA  (2016) 7.5/40 (2015) 8/40 (2014) 6.5/40  (2012) 4/40 | Yes |
| Slurred speech | No | Yes | No | Yes | Yes | Yes | Yes | Yes |
| Saccadic pursuit | No | NA | NA | Yes | Yes | Yes | Yes | Yes |
| **Sensory system** |  |  |  |  |  |  |  |  |
| Vibration sense | Normal | NA | Normal | Abolished | Normal | Mild decreased | Normal | Normal |
| Joint position sense | Normal | NA | Normal |  | Normal | Normal | Normal | Normal |
| Surface sensation | Normal | NA | Normal | Normal | Normal | Normal | Normal | Normal |
| Temperature discrimination | Normal | NA | Normal | Normal | Normal | Normal | Normal | Normal |
| **EMG/ENG** | LMN involvement in Lumbosacral and Cervical region according to revised El Escorial, in Lumbosacral and Cervical and Thoracic regions according to Awaji | Consistent with ALS |  |  |  | Sensory axonal neuropathy | Not performed | Normal NCS sensory sural and motor peroneal nerve; EMG normal triceps, vastus, and tibial muscle |
| **Imaging** | Normal | NA | NA | NA | Cerebellar atrophy | Cortical, corpus callosum, and cerebellar atrophy | Isolated cerebellar atrophy | Not performed |
| **Other symptoms** | No | NA | NA | Ptosis, unable to walk requiring wheelchair | ptosis | ptosis |  | None reported |

**Table 1 (Continued)**

| **Patient’s ID** |  | **6** | **7** | **8** | **9 [published study]** | **10 (VUS in *SPG7*)** | **11 (VUS in *SPG7*)** | **12 (VUS in *AFG3L2*) [published study]** |
| --- | --- | --- | --- | --- | --- | --- | --- | --- |
| **Cohort** |  | NDD (Ataxia) | NDD (Ataxia) | Genesis (Ataxia) | spastic ataxia with optic atrophy and parkinsonism | ALS | Ataxia | Ataxia |
| ***SPG7* mutation**  **(**NM_003119) |  | c.1529C>T:  p.(Ala510Val) | c.1045G>A:  p.(Gly349Ser) | c.1529C>T:  p.(Ala510Val) | c.(376+1_377-1)_(861+1_862-1)del:  p.Glu127SerfsTer2 | c.21_23dup  p.(Leu8dup) | c.1124G>A: p.(Gly375Asp) | c.1529C>T:  p.(Ala510Val) |
| ***AFG3L2* mutation**  (NM_006796) |  | c.2101G>T:  p.(Val701Leu) | c.2065T>C:  p.(Tyr689His) | c.1678del:  p.(Ser560AlafsTer32) | c.1402C>T:  p.(Arg468Cys) | Het CNV (chr18:12362723;ins) | c.1996A>G: p.(Met666Val) | c.2114T>C:  p.(Ile705Thr) |
| **Sex** |  | F | F | M | F | M | F | M |
| **Origin** |  | Germany | Germany | French | Italian | UK | French | Norwegian |
| **Family history and consanguinity** |  | Sporadic, parents second degree cousins | Affected grandmother, father (died at age 46y) and brother | Sporadic, no consanguinity | Sporadic, no consanguinity | Sporadic, no consanguinity | Affected father, no consanguinity | Affected sister and father |
| **Age at onset range (y)** |  | 35-40 | 15-20 | 30-35 | 5-10 | 65-70 | 45-50 | 20-25 |
| **Age at exam range (y)** |  | 55-60 | 70-75 | 40-45 | 25-30 | 65-70 | 55-60 | 45-50 |
| **Pyramidal motor system** |  |  |  |  |  |  |  |  |
| UL/LL spasticity |  | Very mild/none | None/none | None/none | None/yes | Yes/Yes | None/none | None/none |
| UL/LL weakness |  | None/none | None/none | None/none |  | Yes/Yes | None/none |  |
| Tendon reflexes (hyperreflexia) |  | Patellar jerk hypereflexia | Increased lower extremities | Increased biceps and finger |  | Yes | Normal | Decreased reflexes |
| Muscle atrophy |  | None | None | None |  | Yes | None |  |
| Plantar response |  | Flexor | Flexor | Flexor |  |  | Flexor |  |
| Muscle stiffness |  | No | No | No |  | Yes | No |  |
| Ankle clonus |  | No | Yes | No |  | NA | No |  |
| **Gait and balance instability** |  | Yes | Yes | Yes | Yes | NA | Yes | Yes(unsteadiness) |
| **Cognitive deficits** |  | Possible-mild (mild CCAS) | No | No | Mild | No | No | No |
| **Behavioral and psychiatric symptoms** |  | No | No | No |  | No | No |  |
| **Developmental delay** |  | No | No | No |  | NA | No |  |
| **Optic atrophy** |  | No | No |  | Yes | NA |  |  |
| **Decreased visual acuity** |  | No | No | No | Yes | NA | No |  |
| **Bladder dysfunction** |  | Mild (pollakisuria) | No |  |  | NA | No |  |
| **Extrapyramidal motor system** |  |  |  |  |  |  |  |  |
| Brady-/hypokinesia |  | No | No | No | Yes | NA | Yes |  |
| Dystonia |  | No | No | No | Yes | NA | No |  |
| Tremor |  | Yes, cerebellar tremor (intention tremor) |  | No |  | NA | No |  |
| Dyskinesia |  | No | No | No | Yes | NA | No |  |
| Others |  |  | No | No | motor fluctuations | NA | Rigidity |  |
| **Spinocerebellar system** |  |  |  |  |  |  |  |  |
| Oculomotor |  | Yes, horizontal nystagmus, reduced fixation suppression of VOR | Yes, external ophthalmoparesis  Oscillopia  Bilateral ptosis | Yes, horizontal nystagmus  Horizontal Ophthalmoplegia |  | No | Normal |  |
| Dysarthria/dysphagia |  | Moderate/mild-moderate | No | Yes | Mild/none | Yes | Yes/no | Mild/mild |
| Ataxia |  | Yes  SARA  (2017) 15/40  (2016) 14/40  (2014) 15/40  (2012) 13.5/40  (2011) 11/40  (2005) 13/40 | Yes  SARA  (2006) 12/40  (2024) 12.5/40 | Yes  SARA (2015) 11.5/40 (2011) 20/40 | Mild | No | Yes  SARA 5/40 | Yes |
| Slurred speech |  | Yes | Yes | Yes |  | Yes | Yes |  |
| Saccadic pursuit |  | Yes | No | Yes |  | NA | No | Yes |
| **Sensory system** |  |  |  |  |  |  |  |  |
| Vibration sense |  | Normal | Decreased (4/8 ankle) | Decreased |  | NA | Normal | Normal |
| Joint position sense |  | Normal | Normal |  |  | NA | Normal | Normal |
| Surface sensation |  | Normal | Normal | Normal |  | NA | Normal | Normal |
| Temperature discrimination |  | Normal | Normal |  |  | NA | Normal | Normal |
| **EMG/ENG** |  | Normal | Slight reduced amplitudes motor tibial nerve; normal amplitude and velocity of sensory sural nerve |  |  | Consistent with ALS |  | Normal |
| **Imaging** |  | Cerebellar atrophy | Isolated cerebellar atrophy | Cortical and Vermian atrophy | normal | NA | Cerebellar atrophy | Cerebellar atrophy |
| **Other symptoms** |  | OCT thin retina |  | CCFS: 0.945 in 2011  amimia | marked bilateral thinning of retinal fiber layer and ganglion cell layer | NA |  | none |

chr = chromosome; Het = heterozygous; CNV = copy number variation; M = male; F = female; UL = upper limb; LL = lower limb; EMG = electromyography; ENG = electroneurography; LMN = lower motor neuron; SARA = scale for the assessment and rating of ataxia; MOCA = Montreal cognitive assessment; CCAS = cerebellar cognitive affective syndrome; MRI = magnetic resonance imaging; OCT = optical coherence tomography; VOR = vestibulo-ocular reflex. NDD = neurodegenerative disease. NA = not available.
