# Supplementary material for: Digenic inheritance of mutations in SPG7 and AFG3L2 causes motor neuron and cerebellar disorders
Source: BMC Med. 2026 Mar 24;24:283. doi: 10.1186/s12916-026-04805-z (PMC13134353; doi:10.1186/s12916-026-04805-z)
Supplement: Supplementary file 5 — Additional file 5: Fig. 1 - Burden testing results based on the ProjectMinE genome sequencing dataset. The figure derived from Project MinE data browser and shows the exons (orange blocks) in SPG7 and AFG3L2 with the variants (triangles) that were observed in the genome sequencing dataset. [file 12916_2026_4805_MOESM5_ESM.pdf]

SPG7

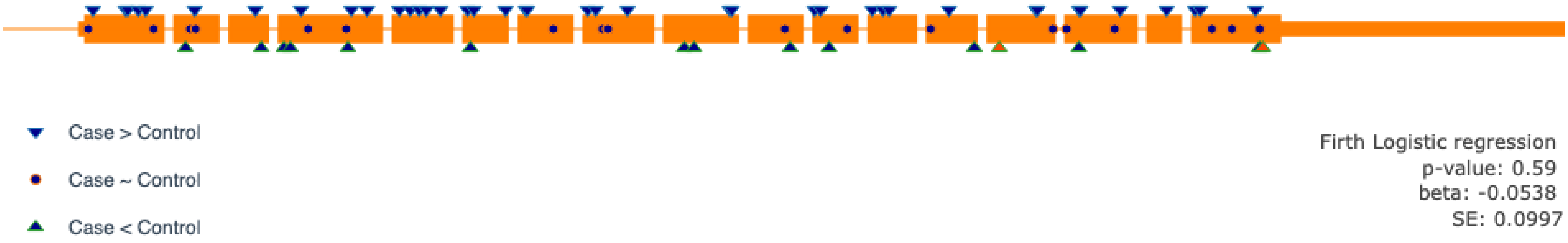
Case > Control
Case ~ Control
Case < Control
Firth Logistic regression
p-value: 0.59
beta: -0.0538
SE: 0.0997

AFG3L2

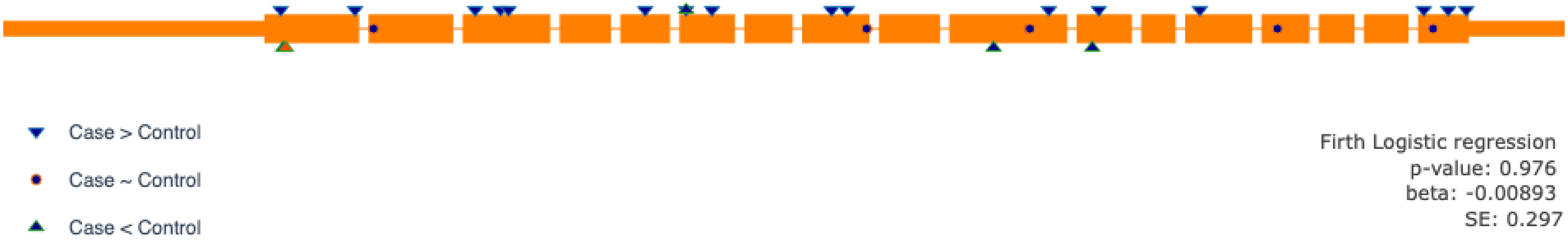
Case > Control
Case ~ Control
Case < Control
Firth Logistic regression
p-value: 0.976
beta: -0.00893
SE: 0.297
